# A Novel pH-dependent Drift Improvement Method for Zirconium Dioxide Gated pH-Ion Sensitive Field Effect Transistors

**DOI:** 10.3390/s100504643

**Published:** 2010-05-05

**Authors:** Kow-Ming Chang, Chih-Tien Chang, Kuo-Yi Chao, Chia-Hung Lin

**Affiliations:** Department of Electronics Engineering, Institute of Electronics, National Chiao Tung University, Hsinchu 30050, Taiwan; E-Mail: kmchang@cc.nctu.edu.tw (K.M.C.); daily.deli@msa.hinet.net (K.Y.C.); ohahaha20@hotmail.com (C.H.L.)

**Keywords:** drift, ISFET, pH-dependent, ZrO_2_

## Abstract

A novel compensation method for Zirconium dioxide gated Ion Sensitive Field Effect Transistors (ISFETs) to improve pH-dependent drift was demonstrated. Through the sequential measurements for both the n-channel and p-channel ISFETs, 75–100% pH-dependent drift could be successfully suppressed for the first seven hours. As a result, a nearly constant drift rate *versus* pH value was obtained, which increases the accuracy of pH measurements. Meanwhile, the drawback of the hyperbolic-like change with time of the common drift behavior for ISFETs was improved. A state-of-the-art integrated scheme adopting this method was also illustrated.

## 1. Introduction

Since the first work for the Ion Sensitive Field Effect Transistor (ISFET) published by P. Bergveld in 1970 [1], numerous chemical and biomedical applications based on ISFET were developed. ISFET has the advantages of rapid response, small size, high input-impedance and low output-impedance, as well as the applicability of semiconductor and integrated-circuit technologies. However, the device instability phenomenon, commonly known as drift, associated with hydrogen ion concentration and time for the ISFET is still one of the critical challenges in developing commercial ISFET-based biomedical sensors. In particular, the high accuracy desired for continuous monitoring in food [2] or biomedical applications requires a tolerable drift rate in pH-ISFETs. The phenomenon and algorithms have been widely discussed by many research groups [3–9]. The summarized factors for drift behaviors are: electric field enhanced ion migration within the gate insulator; electrochemical non-equilibrium conditions at the insulator solution interface; injection of electrons from the electrolyte at strong anodic polarizations to create negative space charge inside the insulator films; and the slow surface effects.

According to the studies of Chou *et al.* [10–14], the pH-independent and pH-dependent drift behaviors were observed on the ISFETs fabricated with different sensing materials and processes, such as hydrogenated amorphous silicon, tin oxide, amorphous tungsten oxide and AlN, *etc.* The pH-independent drift was defined as that the measured potential difference over a period of time in which the ISFET was immersed in the buffer solution of fixed pH value. Proposed solutions to improve pH-independent drift includes the specially designed compensative readout circuits [15], the new device structure with metal oxide as gate contact [16] and the choices of proper sensing films to suppress the influence of the pH-independent drift [17–19]. However, a promising method to deal with the pH-dependent drift has not been available up to date. The pH-dependent drift is obtained by measuring sensors under different pH values buffer solutions and its behavior is quite different from pH-independent drift because of its non-constant characteristics in different pH value electrolytes.

The pH-dependent potential drift is the function of hydrogen ion concentration; as a result, the overall drift - which consists of pH-independent and pH-dependent potential differences - is difficult to be compensated.

Drift behavior of membrane based ISFETs, such as SiO_2_, Si_3_N_4_, Al_2_O_3_ and Ta_2_O_5_ were reported [20–23]. The voltage shift of devices immersed in electrolyte before 10^3^ mins were 3–30 mV for Ta_2_O_5_ [21,22,24], ∼40 mV for Si_3_N_4_ [4,21] and around 50 mV for Al_2_O_3_ [20]. On the other hand, the drift measured after 10^3^ mins were in the range of 0.01–1 mV/ pH. This behavior of hyperbolic-like change with time restricts the measurement accuracy of ISFETs for the first 10^3^ mins.

Eisenman’s theory of ion selectivity has shown that the selectivity is determined by the electrostatic field strength at the ion exchange sites [5]. Surface sites of ZrO_2_ are considered to have strong field strength and therefore should have pH sensitivity. Meanwhile, due to the intrinsic mechanical stability of metal oxide, easy miniaturization and compatibility with CMOS processing, ZrO_2_ is a good candidate for ISFET development. Experimental results show that the ZrO_2_ ISFETs have high pH sensitivity of 57.5 mV/pH, wide linear detecting range of pH 1–13 and low drift of 0.1–0.2 mV/hr after 10^3^ mins operation [25]. It is comparable with other high performance ISFETs, such as Ta_2_O_5_ ISFET [21].

For many applications, the pH-ISFETs are stored in dry environment and always begin each measuring session with a two-point calibration at different pH buffer solutions, such as pH *=* 7.0 and pH *=* 4.01. Those calibrations are always executed in the first hours, which endure the severe potential drift. Meanwhile, the amount of pH-dependent drift is also different, which means that the overall drift cannot be compensated with a simple constant amount. As a result, the accuracy of measurement is restricted or some more complicated compensation circuits are required.

In this study, a novel compensation method by means of sequential measurement of both n-channel and p-channel co-fabricated ZrO_2_ gate ISFETs to improve the pH-dependent drift effect was developed and illustrated.

## 2. Experimental

### 2.1. Device Fabrication

**Figure 1.**
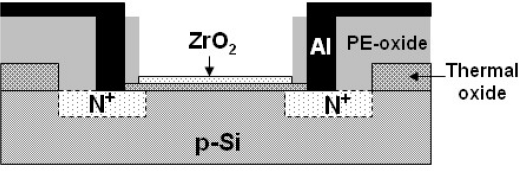
Schematic diagram of ZrO_2_ gate ISFET.

The detailed fabrication procedures and fundamental characteristics of ZrO_2_ gate ISFETs has been reported [18]. Figure 1 shows a schematic diagram of the ZrO_2_ gate ISFET, which was fabricated by the Metal Oxide Semiconductor Field Effect Transistor (MOSFET) technique. The SiO_2_ dielectric FETs were fabricated on both p-type and n-type silicon wafers with (100) orientation accordingly, and their source/drain areas were fabricated with phosphorus/boron ion implantation respectively. A 30-nm-thickness sensing layer of the ZrO_2_ membrane was deposited onto the SiO_2_ gate FET by DC sputtering with 4-inch diameter and 99.99% purity of Zirconium target in oxygen atmosphere The total sputtering pressure was 20 mTorr in the mixed gases Ar and O_2_ for 200 mins while the base pressure was 3 ×10^−6^ Torr, and the RF power was 200 W and the operating frequency 13.56 MHz. Brief manufacturing processes are addressed as follows:

1. Standard RCA clean for 4-inchp-type and n-type silicon wafers
2. Wet oxidation growth for silicon dioxide (600 nm)
3. Defining of Source/Drain areas with mask I and wet etching of silicon dioxide by Buffered Oxide Etching (BOE)
4. Thermal growth of silicon dioxide as screen oxide (30 nm)
5. Phosphorus or boron ions implantation and post annealing at 950 °C
6. Plasma Enhanced Chemical Vapor Deposition (PECVD) deposition of silicon dioxide as passivation layer
7. Defining of contact hole and gate region with mask II and wet etching of silicon dioxide by BOE
8. Dry oxidation of gate oxide (30 nm)
9. DC sputtering of ZrO_2_ (30 nm) and post annealing at 600 °C
10. Defining of gate region and wet etching of oxide by BOE
11. Aluminum sputtering with hard contact mask (500 nm)

### 2.2. Packaging and Measurement

**Figure 2.**
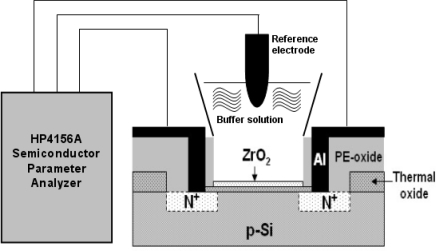
The measurement setup for ZrO_2_ gate ISFETs.

Figure 2 shows the measurement setup and a HP4156A semiconductor parameter analyzer, which was used for measuring the I_DS_−V_GS_ characteristics for the ZrO_2_ gate ISFETs soaked in pH buffer solutions (purchased from R.D.H., Seelze, Germany), where source-drain voltage V_DS_ = 2 V was kept constant. A container was bonded to the gate region of ISFET by using epoxy resin. All the measurements were performed based on a commercial Ag/AgCl glass reference electrode, which was connected to the gate voltage supplier to provide stable bias potential for device operation. The measurements were performed at room temperature of 25 °C, which was kept constant by a temperature control system, and all the setup was placed in a dark box. The measurement of drift was performed and calculated according to the time frame of the first to seventh hours.

## 3. Results and Discussion

### 3.1. pH Sensitivity of n-channel and p-channel ZrO_2_ Gate ISFETs

**Figure 3.**
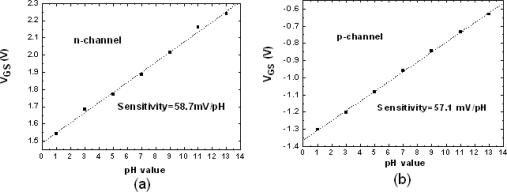
The sensitivity of (a) n-channel and (b) p-channel ZrO_2_ gate ISFETs.

Figure 3 shows the similar sensitivities for both n-channel and p-channel ZrO_2_ gated ISFETs. The measurements were conducted with constant source-drain current and the changes of gate voltage were observed with soaking in various pH buffer solutions. The values were 58.7 and 57.1 mV/pH, respectively. According to the site-biding theory [1], the surface potential established by buffer solution will dominate the change of threshold voltage. The sensitivity has been extensively described in terms of the intrinsic buffer capacity and the differential capacitance.

General expression for the sensitivity of pH-ISFET [26]:

(1) $$\frac{{\Delta \Psi }_{0}}{{\Delta \mathrm{pH}}_{B}}=-2.3\frac{q}{\mathrm{kT}}\alpha$$

with

(2) $$\alpha =\frac{1}{\frac{2.3{\mathrm{kTC}}_{\mathrm{dif}}}{q^{2}\beta_{\text{int}}}+1}$$

Note that *α* is a dimensionless sensitivity parameter with value depending on the intrinsic buffer capacity *β*_int_ and the differential capacitance *C_dif_*. These two factors are determined by the sensing material, processes and electrolyte tested. In this experiment, ZrO_2_ sensing films were fabricated for both n-channel and p-channel ISFETs with the same processes. Consequently, their pH sensitivities should be the same.

### 3.2. Drift Rate of n-type and p-type ZrO2 Gate ISFETs

**Figure 4.**
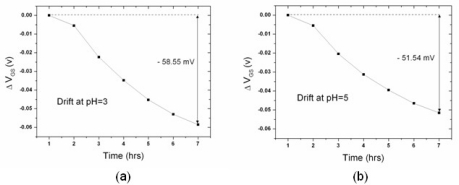

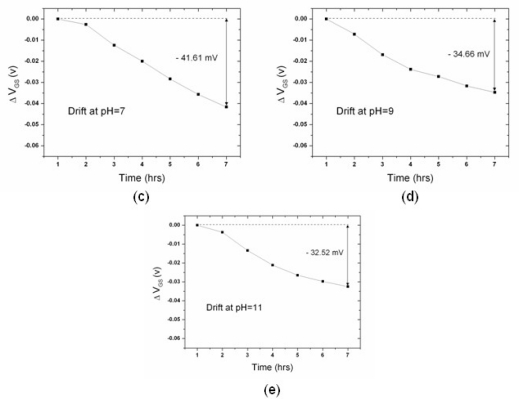
The drift of the n-channel ISFET over the first seven hours at (a) pH3 (b) pH5 (c) pH7 (d) pH9 (e) pH11. The drift over the first seven hours was (a) −58.55mV (b) −51.54mV (c) −41.61mV (d) −34.64mV and (e) −32.52mV, respectively.

**Figure 5.**
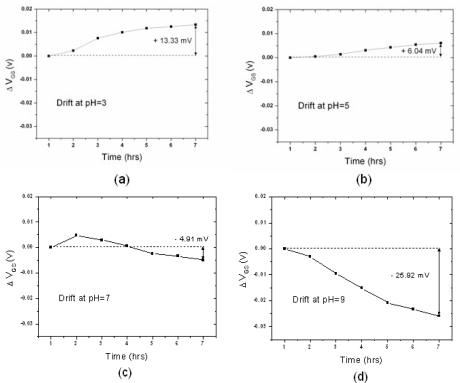

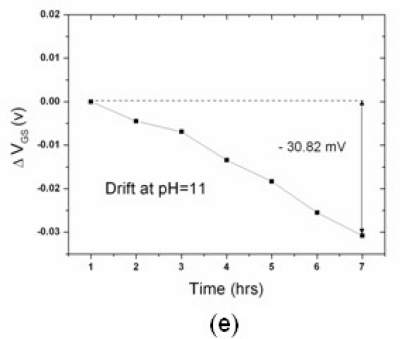
The drift of the p-channel ISFET over the first seven hours at (a) pH3 (b) pH5 (c) pH7 (d) pH9 (e) pH11. The drift over the first seven hours were (a) 13.33mV (b) 6.04mV (c) −4.91mV (d) −25.92mV and (e) −30.82mV, respectively.

**Figure 6.**
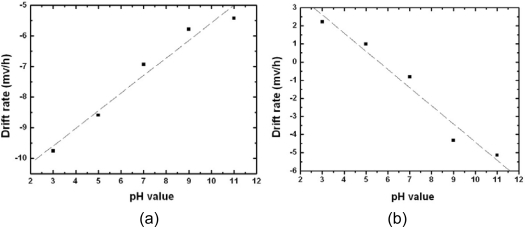
The drift rate of (a) n-channel ISFET and (b) p-channel ISFET for the first seven hours.

Figures 4 and 5 show the drift of the n-channel and p-channel ZrO_2_ ISFET, respectively: the results revealed that the drift is pH-dependent. The potential drifts for pH = 3, 5, 7, 9 and 11 during the time frame of the first seven hours are −58.55, −51.54, −41.61, −34.66 and −32.52 mV, respectively. On the other hand, the values are 13.33, 6.04, −4.91, −25.92 and −30.82 mV for the p-channel ZrO_2_ ISFET. The drift rate for each type of pH-ISFET between hours one to seven was defined as drift voltage divided by time, and the results are shown in Figure 6. The drift rate *versus* different hydrogen concentrations shows the opposite trends for both n-channel and p-channel ISFETs, respectively.

According to the model proposed by Jamasb *et al.* [4], the gate voltage drift can be expressed as below:

(3) ΔVG(t)=−(QD+QI+Qinv)·(ɛn−ɛHLɛnɛHL)xHL(∞){1−exp[−(tτ)β]}

Where *Q_D_* and *Q_inv_* represent the charges stored in the semiconductor depletion layer and the inversion charge, respectively. The signs of *Q_D_* and *Q_inv_* are determined based on the device polarity. *Q_I_* is the effective charge per unit area induced in the semiconductor by the various types of charges that may be present in the insulator, and the magnitude and distribution of the charge entering can be attributed. *ε_n_* and *ε_HL_* represent the dielectric constant of the original sensing layer and the hydrated layer, and *x_HL_* the final thickness of the modified layer. *β* is the dispersion parameter satisfying 0 ≤ *β* ≤ 1. The term xHL(∞){1−exp[−(tτ)β]} represents the chemical hydration dispersive transport, which is an effect that alters the capacitance of dielectrics.

In general, a drift behavior is regarded as a superposition effect of a chemical and an electrical change at the surface of the gate insulator [15]. The chemical change represents the hydration dispersive transport whose degree was determined by both of the hydration species and the material properties of the sensing films. According to the result of previous study [18], the drift rate of the ZrO_2_ film is similar to the result of the Al_2_O_3_ [20], whose extracted value of *x_HL_*(∞) was 13.39 Å. It implies that the ZrO_2_ is also highly effective as transport barrier and the *x_HL_*(∞) of ZrO_2_ should be the same level. Comparing with the thickness of the deposited ZrO_2_ layer (300 Å), it is relatively small. Hence, the hydration transport should be the weak function of Δ*V_G_*(*t*) in this work. On the other hand, when the device is immersed in solutions of different ionic strength, *ε_n_* and *ε_HL_* could be slightly different because of different hydration species. However, since the *x_HL_*(∞) of the ZrO_2_ film is small, the capacitance of hydrated layer is also small and therefore the overall capacitance of the combination of the hydrated and un-hydrated sensing layer was close to the capacitance of the original layer. As a result, $\left(\frac{\varepsilon_{n}-\varepsilon_{\mathrm{HL}}}{\varepsilon_{n}\varepsilon_{\mathrm{HL}}}\right)$ is a weak function of pH and was regarded identical for both n-channel and p-channel ISFETs.

In this work, the trends of the pH-dependent threshold voltage differences were mainly attributed to the overall charge of (*Q_D_* + *Q_I_* + *Q_inv_*). Both *Q_D_* and *Q_inv_* are determined by the nature of the device and are nearly a constant amount under fixed operating conditions. Hence, the pH-dependent drift will be primarily governed by the amount of *Q_I_* in this work. The electrical biasing voltage is a direct effect, which drives in or pulls out the surface and beneath charges of sensing layer, with the result that the effective electrolyte-insulator surface charges density alteres and therefore the threshold voltage changes. Accordingly, the pH-dependent drift can be described as follows:

(4) ΔVG,pH(t)=−QI·(εn−εHLεnεHL)xHL(∞){1−exp[−(tτ)β]}

The dependency of the hydrogen ion concentration was observed. The electrolyte/solid interface charges induced by the higher concentration of the majority species (H^+^ for positive biasing and OH^−^ for negative biasing) were easily influenced by biasing forces and caused the larger potential differences.

### 3.3. Drift Rate and pH Sensitivity of Combined n-channel and p-channel ZrO_2_ Gate ISFETs

**Figure 7.**
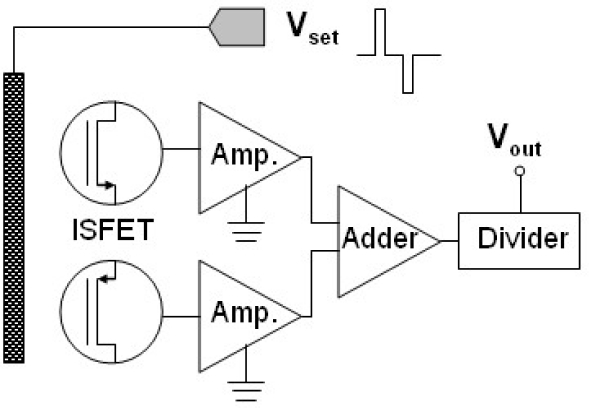
The schematic diagram of the proposed system and measurement method.

Figure 7 shows the schematic diagram of the proposed system and measurement method. The n-channel and p-channel ISFETs can be easily co-fabricated on the same wafer by the standard CMOS processes. While the applied biasing voltage was positive, the n-channel ISFET operated in the linear mode as sensing component and the p-channel ISFET was in cut-off mode. The sensing performances were mainly determined by n-channel ZrO_2_ gate ISFET and the altered threshold voltages should be read out. Similarly, the altered threshold voltages for p-channel ISFETs also should be read out while applying negative biasing voltage. Post signal processing would take the average for both outputs.

**Figure 8.**
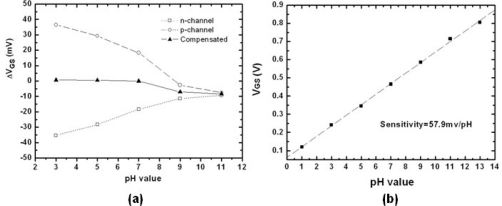
(a) The pH-dependent drift of n-channel, p-channel and corrective ISFETs measured at the seventh hour. (b) The pH sensitivities with the proposed compensation method.

Figure 8 shows the results of the drift and the pH sensitivity of ZrO_2_ gate ISFET measured by the proposed method. The drift from pH 3 to pH 7 was greatly suppressed from 20mV to nearly zero, and drift from pH 7 to pH 11 was also significantly suppressed to less than +/−5mV. According to Equation (4), the pH-dependent drift could be the same but opposite sign for n-channel and p-channel ISFETs; therefore, by measuring both n-channel and p-channel ISFETs sequentially and taking their average values, the elimination of the pH-dependent drift is obtained as Equation (5).

(5) ΔVG,pH(t)=−(QI,n−QI,p)·(εn−εHLεnεHL)xHL(∞){1−exp[−(tτ)β]}≈0

Meanwhile, with the same measurement method, the original pH sensitivity of sole n-channel or p-channel ZrO_2_ ISFET was maintained.

**Figure 9.**
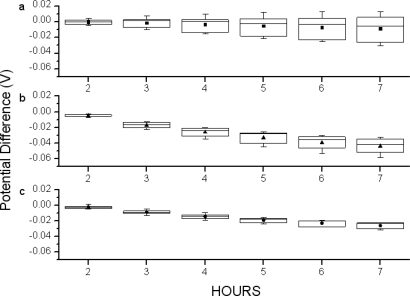
The charts of variations of the drift measured at different pH values from the second to seventh hour for (a) p-channel (b) n-channel ISFETs and (c) the compensation method. The box charts were calculated with the data measured from pH 3–pH 11 at different hours.

Figure 9 shows the variations of the drift measured at different pH values from the second hour to seventh hour for n-channel, p-channel ISFETs and the proposed compensation method. The box charts show the range, standard deviation and mean values of drift measured at pH 3–pH 11. The mean values represent the overall drift and the ranges represent the pH-dependent drift for devices. As observed, the mean values measured at different times for p-channel ISFETs have less potential difference than those of n-channel ISFETs as shown in Figure 9a and b. It seems that p-channel ISFETs have better drift performance. However, their ranges become larger over time while the device is immersed in electrolyte. It revealed that no matter what time the measurements were executed, the exact pH value can not be determined due to the wide range of the potential differences which are pH-dependent. On the contrary, as shown in Figure 9c, the measurement results performed with the proposed compensation method suppressed the ranges significantly. It represents that the pH value for test solutions with proposed method can be determined at any time, and only simple compensation skills are needed. As a result, the pH-dependent drift is significantly improved and the measurement accuracy is increased.

## 4. Conclusions

A proposed scheme and compensation method to improve the pH-dependent drift for ZrO_2_ pH-ISFETs was successfully demonstrated. By the sequential measurement and the post processing of the signals for both n-channel and p-channel ISFETs, the results show that the 75–100% pH-dependent drifts are significantly improved and the nearly constant drift rate *versus* pH value is obtained. Meanwhile, the pH measurement sensitivities are maintained and a practical integrated scheme adopting this method is also illustrated. With the integrated scheme, the calibration accuracy will be greatly improved and no complicated compensation circuit design is required.
